# Shooting the Messenger: The Case of ADHD

**DOI:** 10.1007/s10879-013-9244-x

**Published:** 2013-08-14

**Authors:** Gretchen LeFever Watson, Andrea Powell Arcona, David O. Antonuccio, David Healy

**Affiliations:** 1 Safety and Learning Solutions, Norfolk, VA USA; 2 University of Nevada School of Medicine, Las Vegas, NV USA; 3 Cardiff University, Bangor, Wales UK

**Keywords:** ADHD, Overdiagnosis, Behavioral intervention, Prescription drug abuse, Conflict of interest, Academic freedom, Key opinion leader, Child development, Public health psychology

## Abstract

Medicating ADHD is a controversial subject that was acutely inflamed in 1995 when high rates of ADHD diagnosis and treatment were documented in southeastern Virginia. Psychologists in southeastern Virginia formed a regional school health coalition to implement and evaluate interventions to address the problem. Other professionals with strong ties to the pharmaceutical industry launched ad hominem attacks on the coalition’s research and work. These attacks contributed to the work being terminated in 2005. In the ensuing years, ADHD drug treatment continued to escalate. Today, the national rate of ADHD diagnosis exceeds all reasonable estimates of the disorder’s true prevalence, with 14 % of American children being diagnosed before reaching young adulthood. Notable key opinion leaders continue to claim that there is no cause for concern, but with a message shift from “the prevalence is not too high” to “high prevalence is not too concerning.” This paper provides an object lesson about how innovative research can be derailed to the detriment of sound medical and mental health care of children when industry interests are threatened. Tenure may be the only option for protecting innovative research from specious attacks. The authors offer a summary of the data on ADHD drug treatments, suggest judicious use of such treatments, and add their voices to others who are once again sounding a cautionary alarm.

## Overview

In the mid-1990s, a practicing psychologist in southeastern Virginia, Gretchen LeFever [i.e., Gretchen LeFever Watson], began a program of ADHD research that included epidemiologic surveys. This research documented exceptionally high rates of ADHD diagnosis and drug treatment in her community. With support from Children’s Hospital of The King’s Daughters and Eastern Virginia Medical School (EVMS), LeFever formed a regional school health coalition to improve ADHD treatment. Based on community input, she and her colleagues developed a systematic public health approach to improving the identification and care of children with behavioral problems in the region.

In the course of this work, LeFever was repeatedly attacked for reporting high rates of ADHD diagnosis and treatment. One of the attacks came in the form of an anonymous allegation of scientific misconduct and resulted in the premature termination of LeFever’s work, including her part of a multi-site, multi-million dollar study funded by the Centers for Disease Control and Prevention (CDC) (Lenzer 2005a, b). The anonymous letter alleged that LeFever had falsely reported high rates of ADHD diagnosis to suit her own personal anti-medication agenda. Besides the outright fallaciousness of the anonymous charge, there was then—and is now—ample evidence to support LeFever’s findings regarding high rates of ADHD diagnosis and drug treatment. Nonetheless, the ad hominem attacks had a damaging impact.

First, the attacks contributed to the suppression of a large and unique dataset of risk and protective factors associated with ADHD diagnosis and treatment. Second, the attacks led to the total dismantling of a school health coalition and associated behavioral and public health interventions that showed promise for improving ADHD care.

## LeFever’s “Controversial” Findings

The long-standing ADHD debate had experienced a period of relative quiet until LeFever and colleagues documented high rates of ADHD drug treatment in the mid-1990s. They found that 8–10 % of children in southeastern Virginia, including 17 % of white boys, were being medicated in school for ADHD (LeFever et al. 1999). In one district, 63 % of children who were young for their grade were medicated for ADHD—suggesting a widespread failure to distinguish between disorder and developmentally normal variation. These findings were based on a large-scale and rigorous epidemiologic study of 29,734 children and were published in a prestigious journal—the *American Journal of Public Health (AJPH*) (LeFever et al. 1999). The study drew national and international media attention. LeFever and colleagues conducted a follow-up study that was expanded to include rates of ADHD diagnosis and drug treatment that were not captured by school nurse records (LeFever et al. 2002).

Whether relying on school nurse records or parent report, approximately 9 % of students received a dose of ADHD medication in school during regular school hours (LeFever et al. 2002). However, twice as many children had ADHD according to parent report compared to school nurse records—namely, 17 % of all students in grades two through five and 19 % in grades one through five (LeFever et al. 2003). Among the 17 % of students whose parents said they had ADHD, 84 % had also taken medication for the disorder. Over one-quarter of these students were taking two or more types of psychotropic drugs simultaneously—the most common drug combination involved a psychostimulant like Ritalin or Adderall and an antidepressant like Prozac. Rates were three times as high among boys compared to girls, and twice as high among white children compared to black children. As such, 33 % of white boys (grades 2–5) were purported to have ADHD.

Despite high rates of drug treatment among children identified as having ADHD, their educational outcomes were poor. The students who with reported ADHD were 3–7 times more likely than their peers to experience adverse educational outcomes as defined by repeating a grade, needing special education services, and being expelled and/or suspended. Outcomes were significantly worse among students who had been medicated for ADHD compared to those with ADHD and never medicated for the condition (LeFever et al. 2002). As provocative as these findings might seem, it was ultimately LeFever’s reporting of high rates of drug treatment that were called into question.

## Summary of the Dismantled Public Health Approach to Improving ADHD Care

The School Health Initiative for Education (SHINE) was a regional coalition that LeFever formed in partnership with diverse providers, policy makers, parents, and other community members (LeFever et al. 1999). Through regular meetings that were open to the public, the coalition facilitated and conducted parent, teacher, and provider surveys, focus groups, key informant interviews, and analysis of new and extant databases. Based on an extensive community needs assessment, the coalition identified four major gaps in ADHD care: (1) systematic behavior management, (2) school-provider communication, (3) teacher training and education, and (4) parent training and support (LeFever et al. 2000). LeFever obtained local, state, and federal grant support to implement and evaluate the effectiveness of interventions for each of the community’s self-identified gaps.

With funding from the U.S. Department of Education, LeFever and colleagues implemented a school-wide positive discipline program that resulted in ADHD symptoms decreasing among an elementary school population from the beginning to the end of the school year. This study also documented that teachers who adopted positive classroom management strategies—as evidenced by objective behavioral ratings that achieved a 95 % inter-rater reliability—had students who scored significantly higher on every subject area of the Standards of Learning tests administered to public school students across the state of Virginia (LeFever and Allen 2004; LeFever et al. 2004).

With funding from the Virginia Department of Education, LeFever and colleagues developed a program to facilitate communication—with parental permission—between parents and providers of children who were diagnosed with and/or treated for ADHD. The coalition also developed a single-page ADHD Diagnostic Checklist to remind or apprise parents, school personnel, and providers of the necessary steps to completing a comprehensive ADHD diagnostic assessment process. SHINE members also successfully developed a bill that was passed by the Virginia legislature that prohibited teachers from recommending ADHD medication to parents (LeFever 2008).

Interestingly, parents in the region reported greater satisfaction with behavioral interventions than drug treatment, although their children were far more likely to receive drug treatment than other interventions (LeFever 2008). To expand participation in parent training, LeFever and colleagues used local, state, and federal funding to develop and implement a unique approach to marketing parenting classes. The program—the *A*+ *Behavior Program: Helping Your Student Excel in School and at Home*—experienced unprecedented levels of parent participation. It was so well received that five school districts in southeastern Virginia arranged for their psychologists to receive training and supervision to deliver the program across the region. Some of the participants in this train-the-trainer program were affiliated with a clinical psychology internship that offered a public health psychology tract that LeFever, along with Virginia Beach City Public Schools, spearheaded and led to become the first-ever public health psychology internship in the country—a program that was approved by the American Psychological Association (APA).

These community-oriented interventions appeared to be making a difference. Between 1998 and 2004, southeastern Virginia witnessed a significant (32 %) decrease in the rate of ADHD diagnosis. Among children in first through fifth grade, it declined from a high of 19 % in 1998 to 13 % in 2004 (LeFever and Allen 2004). Unfortunately, the APA-approved internship in public health psychology, the SHINE Coalition, and projects described above were terminated as a result of unjustified attacks on LeFever’s research findings.

## The Allegation of Scientific Misconduct

Although LeFever was never allowed to see the anonymous and type-written allegation of scientific misconduct that was lodged against her, she was informed that the gist of the allegation was that she had intentionally inflated rates of ADHD diagnosis and treatment to suit her own anti-medication agenda. Reportedly, the allegation made reference to a figure of 17 % that was reported in one of her publications. The figure of 17 % appears in more than one publication and it was never made clear which publication was being called into question. The anonymous complaint also reportedly alleged that LeFever was conducting research without proper consent. All of LeFever’s research had been properly submitted to and reviewed by the Institutional Review Board at EVMS where she was employed. For the record, LeFever did not have an anti-medication agenda and had been involved with the referral of many children for medication evaluations.

As outlined below, the allegation and corollary repercussions led the medical school to threaten to fire LeFever. Thirty-nine psychiatrists and psychologists responded in outrage by signing a petition expressing to the EVMS Dean that LeFever should have been commended and promoted for having the courage to be among the first to sound the alarm (Lenzer 2005b). LeFever was eventually cleared of all charges of scientific misconduct, honored with a sabbatical, and supported by the Dean for promotion to Full Professor (Lenzer 2005a). Nonetheless, the “chopping block” experience “derailed” LeFever’s career (Whitaker 2010).

## Orchestrated Attacks on Health Researchers

Attacks on health researchers who are at odds with the pharmaceutical industry are not new (Healy 2002). As noted in a *New England Journal of Medicine* article (Deyo et al. 1997), there is a pattern to such attacks. Individuals who initiate them have financial interests in maintaining the legitimacy of a medical model of illness or a particular treatment. They have used allegations of scientific misconduct and the media, as well administrative and legal assaults, to malign the integrity of researchers and their findings—all of which LeFever experienced. The authors noted that in one documented case, a trial lawyer sponsored a workshop promoting the use of allegations of scientific misconduct as a weapon for disputing unwelcome research findings.

Regarding ADHD research, LeFever is not the only scientist who experienced malicious attacks. Nadine Lambert, a renowned school psychologist, endured a similar experience. Lambert created a stir when she reported to the National Institutes of Health that children who were treated with stimulant medications like Ritalin started smoking cigarettes earlier, smoked more heavily, and were more likely as adults to abuse cocaine than were those not taking ADHD medications (Lambert 1999). These results were based on the culmination of a 30-year longitudinal study of 492 children—about half of which had been diagnosed with ADHD. Like LeFever, Lambert was falsely accused of scientific misconduct (Diller 2005). Although eventually cleared, the allegation derailed her research—research that was never resurrected before Lambert died in a tragic 2006 head-on collision with a truck.

These are not isolated incidents. There are many other examples of scientists who have been harassed, bullied, or had their jobs threatened because they stood up to industry and refused to let data be buried our spun (Blumsohn 2006; Healy 2002, 2008; Marks et al. 1993; Monbiot 2002; Nathan and Weatherall 2002; Thompson et al. 2001).

## Industry Support for LeFever’s Most Outspoken National Critic

Over the years, LeFever’s most outspoken national critic has been clinical psychologist Russell Barkley, Ph.D. Barkley is known in the field as a key opinion leader. An industry-sponsored ‘opinion leader’, or ‘key opinion leader’ refers to someone who is an active media user and who interprets the meaning of subject-specific information for the broader public (Elliott 2010). The pharmaceutical industry makes liberal use of key opinion leaders, and Barkley may be the best-known ADHD key opinion leader the industry has courted. Although such individuals can be patient advocates, they run the risk of becoming a marketing spokesperson for the industry—unwittingly or otherwise.

Barkley repeatedly criticized LeFever based on what he has expressed to be the unpalatable nature of her findings rather than offering substantive criticism of her research methodology or data analysis. Given Barkley’s prominence as an established and leading scientist and clinician in the field of ADHD, his comments about exaggerated emerging prevalence trends carried weight. It is unclear how many people knew then (or realize now) the significance of the fact that a sizeable proportion of Barkley’s taxable income came from the pharmaceutical industry. Barkley’s own website once showed, for example, that approximately 8 % of his taxable income came from Eli Lilly alone. Eli Lilly manufactures Strattera, a commonly prescribed medication for ADHD. Other income categories that were explicitly tied to the pharmaceutical industry accounted for approximately 19 % of his income (LeFever 2007). Like other ADHD opinion leaders, Barkley also has had extensive support from Children and Adults with Attention-Deficit/Hyperactivity Disorders (CHADD)—a prominent advocacy group that is supported with funds from the manufacturers of ADHD medications.

## Attacks on LeFever’s Research (1996–2004)

Barkley’s criticism of LeFever began as early as 1996 when LeFever first shared her findings of high ADHD drug treatment rates (findings that would eventually be published in *AJPH)* with leading healthcare providers in southeastern Virginia. In response to growing concerns about ADHD treatment trends, EVMS pediatric faculty invited Barkley to address the topic as the keynote speaker for the region’s annual pediatric meeting. The annual professional meeting included a talk that was open to the public. As a recognized authority on a topic of great concern to the general public, Barkley drew a huge crowd for his public address. Barkley contended that ADHD was a genetic disorder that was not being overdiagnosed or overtreated, but that most children with ADHD were not receiving medication when they should have been (Tennant 1996).

Not long thereafter, LeFever’s supervisor—a pediatrician whose medical practice involved extensive ADHD referrals—attempted to intimidate LeFever by abruptly cancelling all her patients, informing LeFever’s coworkers that she no longer worked at the hospital, and presenting her with an unsanctioned request that she resign immediately. Having caught wind of the event, the medical school leadership required the physician to provide LeFever with a written and verbal apology and invite her back to practice with him. Instead, she accepted an offer from the medical school’s Center for Pediatric Research to assume a full-time research faculty position. Thus began the campaign to discredit and silence LeFever.

Barkley returned to Hampton Roads in 2000 to debate LeFever on the topic of medicating ADHD. She was allotted 15 min to present; Barkley was given 45 min. Barkley began his talk by asserting that LeFever was not a scientist, summarily dismissing her research out of hand. Just a few months later, Barkley again attempted to discredit LeFever when LeFever was invited to join an APA panel discussion on ADHD treatment solutions. The panel was comprised of experts who had been working in the field of ADHD much longer than LeFever, including Drs. Peter Jensen who had recently stepped down from a lead position at the National Institutes of Health, William Pelham (MTA investigator and Professor at SUNY Buffalo), Larry Diller (pediatrician and author of *Running on Ritalin*), Charles Cunningham (Professor at McMaster University and ADHD researcher), and, of course, Barkley (perhaps the most widely recognized name in the ADHD field). What ensued was not so much of a planned discussion of treatment solutions but, rather, a debate about ADHD drug treatment trends.

The following year, in 2002, Barkley published a paper declaring that individuals who questioned the rising rates of ADHD diagnosis and treatment represented “social critics and fringe doctors.” He invited at least 85 psychologists and psychiatrists to co-sign the paper. In the paper, which he titled the International Consensus Statement on ADHD, Barkley dismissed people who expressed a point of view that was contrary to his as “tantamount to declaring the earth [Earth] flat, the laws of gravity as debatable, and the periodic table a fraud” (Barkley and 84 other behavioral scientists 2002, p. 3).

The following year (2003) another international group of mental health professionals responded by publishing a critique of Barkley’s statement (Timimi et al. 2004). Their critique began by asking why a group of eminent psychiatrists and psychologists would produce a consensus statement that sought to forestall debate on the merits of widespread ADHD diagnosis and drug treatment. They asserted that shutting down debate prematurely was completely counter to the spirit and practice of science and reminded readers that one generation’s most cherished therapeutic ideas and practices are often repudiated by the next generation, but not without leaving countless victims in their wake. This critique referenced LeFever’s *AJPH* study findings as evidence against Barkley’s ongoing assertion that less than half the children who need ADHD medication are receiving medications (Timimi et al. 2004). Barkley responded strongly with a published rebuttal (Barkley et al. 2004).

## A Call for Investigating LeFever’s Findings through the Academic Press (March 2004)

Barkley’s rebuttal to the Timimi et al. critique of his consensus on ADHD (Barkley et al. 2004) failed to cite numerous studies that supposedly supported his argument. The one study that he did choose to identify was Tim Tjersland’s doctoral dissertation. This dissertation study was methodologically flawed and remains unpublished nearly a decade after completion (Tjersland 2004). Barkley misrepresented the dissertation research as a replication study of LeFever’s AJPH research and inaccurately reported that it found prevalence rates near three percent in southeastern Virginia. Not only was Tjersland’s study not a true replication study, it did not produce the findings that Barkley described. If anything, Tjersland’s results corroborated LeFever’s findings. Of note, Barkley himself was part of Tjersland’s dissertation committee. Based on this methodologically flawed and unpublished study, Barkley claimed that LeFever’s findings from multiple peer-reviewed and published studies were so questionable that they “deserve investigation” (Barkley et al. 2004, p. 68).

## Investigative Call was Answered (April 2004)

Within weeks of Barkley’s call for an investigation of LeFever’s findings, someone submitted an anonymous complaint about LeFever’s work to EVMS (i.e., the complaint described above). In response, EVMS conducted an internal investigation of LeFever’s past and current research. Against EVMS policy and common protocol for investigation of allegations of scientific misconduct, the medical school confirmed to the media that LeFever was under investigation.

Before LeFever was aware of the allegation of misconduct, the medical school had conducted a review of more than a decade of her research. The process identified that there might be a typo between the wording of a survey item and the manner in which the survey item was described in the appendix of a published article. Until the reported typo was brought to LeFever’s attention, neither she nor any of her three co-authors had ever noticed the discrepancy.

## Definition of Scientific Misconduct

Scientific or research misconduct is defined as fabrication or falsification of research, plagiarism, or other practices that deviate significantly from what is commonly accepted within the scientific community research. It does not pertain to honest error or differences in interpretations or judgments of data (Office of Research Integrity 1997, p. 3).

## LeFever Cleared of Misconduct Charges (July 2004)

LeFever felt that it was important to explore how the identified error had occurred and what, if any, impact it had on reported outcomes. She researched reasons for the discrepancy and detailed them in a written report that was submitted to the EVMS scientific misconduct committee that had been convened for her case. She met with the committee and medical school attorneys for several hours of testimony—all of which was tape-recorded. Later that day, LeFever was informed that the committee had unanimously determined that there was no evidence of scientific misconduct and that the typo appeared to be an honest error that had no impact on research conclusions. No finding of misconduct was ever reported to the Office of Human Research Protection, as would have been required if LeFever had violated consent procedures.

The EVMS committee did ask LeFever to inform the journal where the study with the typo had been published to disclose the error. She did so forthwith and in writing. The journal’s Editor determined that the typo was too minor to warrant any corrective action. The matter should have been dropped, but instead inquiries about consent procedures and reported findings escalated.

## Reporter-Generated “Evidence” of “Misconduct”

Although the journal determined that the error in LeFever’s publication was too minor to warrant a corrective statement, the Editor subsequently contacted LeFever to share that a reporter (Bill Sizemore of *The Virginian Pilot)* had repeatedly asked her to publish the error statement. Phelps lamented to LeFever that she and her co-Editor, who also felt that the error was too minor to warrant any action, finally decided to turn the matter over to the publishing house. The journal’s publishing house decided for the sake of public relations/business reasons—not for reasons pertaining to scientific integrity—that they would publish a brief error statement in the next issue of the journal (Phelps, personal communication, January 2005; April 2007), which appeared in a subsequent issue (LeFever et al. 2005).

## Relentless and Prejudiced External Interference (April 2004–January 2005)

LeFever endured months of waiting for her name to be cleared and research to be re-approved for continuation. EVMS eventually cleared her of all charges of scientific misconduct and re-approved her research for continuation. However, that LeFever was under investigation became common knowledge among the medical school staff and faculty, community collaborators, city leaders, and the press. The day after LeFever’s research was finally re-approved for continuation, the approval was rescinded. Apparently, this news also leaked out, and more complaints about her research reportedly surfaced. LeFever never learned exactly who complained about what, but she was informed that all the concerns were investigated and dismissed as unfounded.

Eventually, a “research ethicist” by the name of Felix Gyi, M.D. who had been communicating with EVMS was asked to express his opinion directly to LeFever during a conference call with her and EVMS administrators and attorneys. Gyi was CEO of Chesapeake Research Review, which is a for-profit company whose primary clients are major pharmaceutical companies and universities conducting research funded by the pharmaceutical industry. Chesapeake Research Review was involved with at least one ADHD drug trial involving both EVMS faculty and Barkley. Gyi asserted that LeFever’s CDC-funded research represented more than minimal risk to subjects and, therefore, proper consent procedures had not been used. In response, EVMS halted LeFever’s work.

On or about December 9, 2004, a Virginia Beach school district official reportedly complained to the EVMS Dean that LeFever had misled her about procedures to obtain parental consent for her CDC-funded epidemiologic survey study that was underway in area school districts. This allegation was false and wholly unsupported by the record. Fear on the part of school officials of possible legal action and press scrutiny apparently created an atmosphere in which the self-preservation instinct overcame solid factual analysis of what transpired. The unsubstantiated claim by a school official that she had been misled about LeFever’s research protocol and consent procedures was the straw that broke the camel’s back. The ordeal and looming threat of a scandalous newspaper expose about local ADHD research had the potential to become a public relations nightmare for the medical school and collaborating school districts. On December 14, 2004, the very newly appointed and Interim Dean of the medical school (under advisement from attorneys who may not have appreciated the full academic impact of their legal positions) permanently terminated LeFever’s research, placed her on administrative leave (Lenzer 2005b) and wrote to public school officials promising that the study data would never be used for any purpose.

## LeFever was Defamed in the Public Press (January 2005)

A long-anticipated newspaper “expose” of LeFever’s “wrongdoing” was finally published on January 25, 2005 (Sizemore 2005). The reporter failed to mention his role in pressuring the journal to publish the statement despite the fact that the editor had determined that it was unnecessary and was inconsequential to the study’s findings and conclusions. This public relations fiasco effectively extinguished any chances of LeFever re-kindling relationships that were vital to the continuation and success of her work.

As such, the article brought an end to ADHD research and community-based interventions in southeastern Virginia—work that might have served as a model for improving mental health care in other communities dealing with high rates of diagnosis and drug treatment. The newspaper story quoted a local psychologist with ties to CHADD who was concerned that LeFever’s work frightened parents away from seeking appropriate treatment for their children and Barkley who described LeFever’s findings as “highly suspicious” (Sizemore 2005). The net effect was that a decade of LeFever’s research and community work described earlier was dismantled, and the ADHD debate was significantly quieted in the ensuing years.

## Landslide Victory for Big Pharma

The pharmaceutical industry and its key opinion leaders were apparently successful in quelling our nation’s concerns about high rates of ADHD drug treatment. Since 2005, we have witnessed a message shift from “the rate of ADHD drug treatment is not too high” to “a high rate of ADHD drug treatment is not too concerning” (Scudder 2011).

In the years following the shutdown of LeFever’s work, CDC reports documented continual increases in the rate of ADHD diagnosis and drug treatment (CDC 2010; Sondik et al. 2012). The CDC has reported that 11 % of American children are currently diagnosed with ADHD (CDC 2013) and that 14 % will receive a diagnosis of ADHD before the end of childhood (i.e., by 15–17 years of age) (CDC 2010). This national rate of ADHD diagnosis is now up to 50 % higher than the rate that was reported by LeFever in *AJPH* over a decade ago (8–10 %)—the rate that once sparked national and international debate. Calculations based on 2010 CDC data also suggest that nearly 20 % of American boys are now diagnosed with ADHD by 15–17 years of age (CDC 2013). Similar trends were found in 2011 (Sondik et al. 2012). These reports and the most recent CDC data (CDC 2013) also documented that ADHD diagnosis and treatment rates continue to vary widely by geographic region as documented more than a decade ago (e.g., Morrow et al. 1998). The most recent CDC data indicate that ADHD is now the most prevalent mental health diagnosis among children 3–17 years of age, with rates that vary substantially by state (from a low of 5.6 % in Nevada to a high of 15.6 % in North Carolina) (CDC 2013). Due to within state variation, some communities probably have experienced rates of diagnosis that are notably higher than the national average of 14 % or the state high of 15.6 %. Yet, there has been limited professional or public outcry about the ever-rising rate of ADHD diagnosis and drug treatment.

## Rise of Ritalin is Replaced by the Rise of Risperdal

More disturbing than the high level of psychostimulant drug treatment is the growing numbers of children who are being prescribed an ever-widening formulary of powerful psychotropic drugs to treat ADHD. For example, prescriptions for antidepressants (which are often added to psychostimulant treatment regimens for children with ADHD have increased over 400 % in recent years (Pratt et al. 2011). Antipsychotic drug prescriptions—prescriptions for drugs like Risperdal that historically have been reserved for treatment of adults with schizophrenia and other psychotic disorders—increased eight-fold among children during the last two decades (1993-2009) (Olfson et al. 2012) with the nation’s poorest children among the most common recipients (e.g., Zito et al. 2013). Data collected between 2005 and 2009 reveal that, of all children’s physician office visits, almost 2 % result in the prescription of an antipsychotic drug. The rate is almost 4 % for adolescents and skyrockets to 30 % when the visit involves a psychiatrist (Olfson et al. 2012). Between 2005 and 2007, the state of Florida witnessed a 250 % increase in the prescription of antipsychotic drugs for children (Farley 2007). This included 1,100 Medicaid children as young as 3 years of age. Many such prescriptions are specifically for children carrying a diagnosis of ADHD (Matone et al. 2012); others are likely prescribed for iatrogenic effects of ADHD drug treatment (Whitaker 2010).

As alarming as these trends are in and of themselves, there is yet another (and more disturbing) layer to the story of ADHD treatment trends. Many children are being treated with more than one psychotropic drug at a time (Mojtabai and Olfson 2010; Zonfrillo et al. 2005). If one drug is dangerous on its own, certainly combining two drugs increases the risk of harm exponentially. There is little research clearly investigating these effects, but emerging data suggests significant risk (Mojtabai and Olfson 2010; Zonfrillo et al. 2005). The documented rate of diagnosis is now so high and the use of a host of psychotropic drugs so prevalent that it should raise concern among all healthcare professionals, child advocates, and parents.

## What’s the Harm of Casually Diagnosing and Aggressively Medicating ADHD?

The more a drug is prescribed, the more it is available for diversion and abuse. Research has shown that as early as 15 years ago in some communities, up to 16 % of elementary through high school students had been approached by classmates to share or sell their ADHD medications (Musser et al. 1998); 5 years later, up to 23 % of middle and high school students had been approached (McCabe et al. 2004). By 2006, 34 % of students attending a large southeastern college reported using ADHD drugs illegally (DeSantis et al. 2008). The ubiquitous availability of ADHD medications on high school and college campuses has led many teens and young adults to perceive drugs like Adderall to be benign for academic enhancement (e.g., Desantis and Hane 2010; DeSantis et al. 2008, 2010; Wish et al. 2005). They also view these drugs as relatively safe for recreational use and freely mix them with alcohol (e.g., Desantis and Hane 2010; DeSantis et al. 2008)—a potentially lethal combination. Growing numbers of students are now sharing, swapping, stealing, and abusing ADHD medications. Not surprisingly, increases in ADHD prescriptions have been associated with a parallel escalation in abuse of ADHD drugs.

Analysis of national poison control data for 1998–2005 showed a sharp increase in the number of children between 13 and 19 years of age who were reported to poison control due to ADHD medication abuse—an increase that was disproportionately higher than drug abuse generally or for teen substance abuse in particular (Setlik et al. 2009). The severity of cases also increased over time, particularly for amphetamines (e.g., Adderall) compared to methylphenidates (e.g., Ritalin) (Substance Abuse and Mental Health Services Administration 2013). Nationwide, emergency department visits for adverse reactions to prescription use of such drugs as well as illicit use of such drugs also increased between 2005 and 2009 (National Institute of Drug Abuse 2011).

Even when used as prescribed, ADHD drug treatment often brings with it adverse side effects. The side effects may include undermining of an individual’s motivation to take other steps to address behavioral issues, deflating of one’s sense of self-efficacy, and/or flattening of one’s affect or natural exuberance. ADHD drugs also carry the potential for side effects such as sleep disturbance and growth suppression, as well as more serious side effects like elevated risk for drug dependence, psychosis, cardiac arrest, and violence against oneself or others (Moore et al. 2010). Between 2004 and 2011, there were more than 11,000 cases of psychotropic drug side effects related to violent actions reported to the FDA’s MedWatch system. And these are only the side effects that are reported to the FDA, which constitute only an estimated 1–10 % of actual occurrences.

Despite the fact the FDA requires that mainstream ADHD stimulant drugs carry a “black box” warning label noting the drugs’ risks for addiction, psychosis, and cardiac arrest, many professionals, parents, and patients underestimate the power of prescription pills due to “successful” marketing campaigns (e.g., Lacasse and Leo 2009). Popular media have also aided the pharmaceutical industry’s overselling of the benefits of ADHD drugs (Gonon et al. 2012). Findings that emerged from our country’s epic MTA Study (Kollins et al. 2006; Richters et al. 1995)—a large-scale, longitudinal study funded by the National Institutes of Health—showed that: (a) the benefits of drug treatment (even carefully monitored drug treatment) faded over time while the benefits of behavioral treatment endured (Pelham and Fabiano 2008); (b) drug treatment was not effective as delivered as part of routine community care (Greenhill et al. 2001); (c) drug treatments did not result in better academic outcomes (Molina et al. 2009); (d) over time, behavioral interventions were more effective than drug treatment (Pelham and Fabiano 2008) and (e) when behavioral interventions were implemented *prior* to the initiation of drug treatment, 75 % of children fully resolved their ADHD symptoms (Pelham 1999). Yet, the pervasive public message was that ADHD drugs work and should be the first line of defense (Pelham and Fabiano 2008). In fact—as is also the case with depression (Antonuccio 2008; Antonuccio et al. 1995, 1999, 2002)—the scientific evidence indicates that psychosocial interventions for ADHD are at least as effective as ADHD drug treatment when long-term outcome is considered (for a review, see Pelham and Fabiano 2008).

In addition to overselling drug benefits, the pharmaceutical industry minimizes drug risks (Lacasse and Leo 2009). Individuals who experience the side effects are portrayed as biologically predisposed and/or otherwise vulnerable to emotional breakdowns (e.g., Sizemore 2012). Such generalizations can lull clinicians and parents into a false sense of security and belief that the side effects will not occur for otherwise “normal” children.

## Summary

ADHD experts with ties to the pharmaceutical industry and/or CHADD repeatedly launched ad hominem attacks on work by a psychologist whose research findings conflicted with drug industry interests. These attacks ultimately led to a decade of significant ADHD research and community-based interventions being mischaracterized in professional venues and media outlets. The net effect was that research on psychosocial interventions that also raised questions about the effectiveness of ADHD drug treatment was terminated and study findings were suppressed. This helped pave the way for continued escalation and expansion of ADHD diagnosis and drug treatment among American children, youth, and adults. The rate of ADHD diagnosis now exceeds all reasonable estimates of the true prevalence of the disorder. As a consequence, ADHD drugs are readily available on American high school and college campuses where they are increasingly abused with serious and sometimes lethal consequences.

## A Professional Call to Arms

With mounting evidence of serious risks associated with widespread use of psychotropic drugs, the case of the attack on LeFever and suppression of the data she generated can serve as a wake-up call for mental health professionals. This case provides an object lesson about why tenure is so important in protecting academic freedom. Without tenure, the risks to the independent academician can be great if billions of industry dollars may be threatened (Antonuccio et al. 2003). It is up to independent scientists to address the inflation of benefits of drug therapies and the minimization of risk (Healy 2002, 2008; Leo and Cohen 2003). Prominent Psychiatrist Allen Frances and Psychologist Alan Stroufe have recently again sounded the alarm about ADHD being overdiagnosed, medications being overused, and the lack of long-term benefit from these medications (Frances 2013; Stroufe 2012). The authors would like to add their voices to their call.
